# Expenditures for the care of HIV-infected patients in rural areas in China's antiretroviral therapy programs

**DOI:** 10.1186/1741-7015-9-6

**Published:** 2011-01-17

**Authors:** Feng Zhou, Gerald F Kominski, Han-Zhu Qian, Jiansheng Wang, Song Duan, Zhiwei Guo, Xinping Zhao

**Affiliations:** 1 State Key Laboratory for Molecular Virology and Genetic Engineering, Institute of Pathogen Biology, Chinese Academy of Medical Science, Peking Union Medical College, Beijing, PR China; 2 Center for Health Policy Research, University of California, Los Angeles, California, USA; 3 Institute for Global Health and Division of Epidemiology, Vanderbilt University School of Medicine, Nashville, Tennessee, USA; 4 Center for Public Health Surveillance and Information Service, Chinese Center for Disease Control and Prevention, Beijing, PR China; 5 Center for Disease Control, Dehong Dai and Jingpo Autonomous Region, Yunnan Province, PR China; 6 School of Public Health, Peking Union Medical College, Beijing, PR China; 7 Wenxi County Hospital, Wenxi County, Shanxi Province, PR China

## Abstract

**Background:**

The Chinese government has provided health services to those infected by the human immunodeficiency virus (HIV) under the acquired immunodeficiency syndrome (AIDS) care policy since 2003. Detailed research on the actual expenditures and costs for providing care to patients with AIDS is needed for future financial planning of AIDS health care services and possible reform of HIV/AIDS-related policy. The purpose of the current study was to determine the actual expenditures and factors influencing costs for untreated AIDS patients in a rural area of China after initiating highly active antiretroviral therapy (HAART) under the national Free Care Program (China CARES).

**Methods:**

A retrospective cohort study was conducted in Yunnan and Shanxi Provinces, where HAART and all medical care are provided free to HIV-positive patients. Health expenditures and costs in the first treatment year were collected from medical records and prescriptions at local hospitals between January and June 2007. Multivariate linear regression was used to determine the factors associated with the actual expenditures in the first antiretroviral (ARV) treatment year.

**Results:**

Five ARV regimens are commonly used in China CARES: zidovudine (AZT) + lamivudine (3TC) + nevirapine (NVP), stavudine (D4T) + 3TC + efavirenz (EFV), D4T + 3TC + NVP, didanosine (DDI) + 3TC + NVP and combivir + EFV. The mean annual expenditure per person for ARV medications was US$2,242 (US$1 = 7 Chinese Yuan (CNY)) among 276 participants. The total costs for treating all adverse drug events (ADEs) and opportunistic infections (OIs) were US$29,703 and US$23,031, respectively. The expenses for treatment of peripheral neuritis and cytomegalovirus (CMV) infections were the highest among those patients with ADEs and OIs, respectively. On the basis of multivariate linear regression, CD4 cell counts (100-199 cells/μL versus <100 cells/μL, *P *= 0.02; and ≥200 cells/μL versus <100 cells/μL, *P *< 0.004), residence in Mangshi County (*P *< 0.0001), ADEs (*P *= 0.04) and OIs (*P *= 0.02) were significantly associated with total expenditures in the first ARV treatment year.

**Conclusions:**

This is the first study to determine the actual costs of HIV treatment in rural areas of China. Costs for ARV drugs represented the major portion of HIV medical expenditures. Initiating HAART in patients with higher CD4 cell count levels is likely to reduce treatment expenses for ADEs and OIs in patients with AIDS.

## Background

According to the most recent estimates by the Ministry of Health and the Joint United Nations Program on HIV/AIDS (UNAIDS), China had 560,000-920,000 people living with human immunodeficiency virus (HIV) and 97,000-112,000 people living with acquired immunodeficiency syndrome (AIDS) as of the end of 2009. A total of 320,000 HIV/AIDS cases have been reported from the national infectious disease surveillance system from 1985 to 2009 [1]. The first wave of AIDS cases was reported in China around 2002, and the majority of these AIDS patients contracted the virus in the 1990s, through either the injection of drugs or plasma donation. In response, the Chinese government launched the China Comprehensive AIDS Response (CARES) in 2003, a community-based HIV treatment, care and prevention program that provides free HIV treatment, including antiretroviral (ARV) therapy and treatment of opportunistic infections (OIs) to the indigent in urban areas and to everyone in rural areas [2].

China CARES provides first-line ARV regimens, including two nucleoside reverse transcriptase inhibitors (NRTIs) and one non-nucleoside reverse transcriptase inhibitor (NNRTI), the generics of which are produced in China. According to national guidelines, ARV treatment is provided by designated infectious disease hospitals, and patients in rural areas in some provinces may go to village clinics to receive counseling and treatment for OIs. Staff from the local Centers for Diseases Control and Prevention (CDC) provides education on ARV treatment compliance and disease prevention intervention. An estimated 97% of AIDS patients (>50,000) who initiated highly active antiretroviral therapy (HAART) in China received free treatment through this national program by 2008; 3% of these patients received treatment via self-pay or through some nongovernmental organization-sponsored treatment programs that may use different regimens [3,4]. The central government increased funding for HIV prevention and treatment from US$56 million in 2003 to US$142 million in 2008, while local governments increased funding from US$14 million to US$86 million in the same period [1]. Recent analyses of national data from China CARES have shown that it significantly decreased mortality among HIV-infected patients from 27 deaths per 100 person-years before treatment to 4-5 deaths per 100 person-years after 6 months of HAART [3,5].

The Comprehensive International Program for Research on AIDS (CIPRA) is an initiative sponsored by the U.S. National Institutes of Health (NIH). China CIPRA comprises five interrelated projects. The overall goal of China CIPRA is to strengthen the existing HIV research infrastructure in China and to build the capacity to conduct multidisciplinary research to explore promising strategies for HIV prevention, treatment and control. Subproject 4 is "A Feasibility Study of Lamivudine/Zidovudine (3TC/ZDV) plus Efavirenz (EFV) as Initial Therapy of HIV-1-infected Patients in Rural Areas of China." The project was conducted between June 2005 and June 2007. The primary objective of Subproject 4 was to determine the efficacy and safety of a potent combination ARV regimen (3TC/ZDV + EFV) for the treatment of HIV-1-infected patients in a rural area of China. China CIPRA covered all of the costs for patients during the research period.

However, there are no publications on the expenditures and cost-effectiveness of treating HIV-infected patients in China. Considering the fact that treatment providers, targeted clients and economic assistance policies may vary across different areas, the actual expenditures of this program are largely unknown. Such information is needed for future financial planning and possible reform of AIDS-related policies. Therefore, the current study determined the expenditures associated with two ARV treatment programs in rural China.

## Methods

### Study Settings

This retrospective cohort study was conducted in two geographical areas: Dehong Prefecture of Yunnan Province in southwestern China and Wenxi County of Shanxi Province in central China. Enrollment criteria and follow-up periods were slightly different at the two study sites. The HIV/AIDS epidemic in Dehong Prefecture primarily consists of injecting drug users (IDUs) and female sex workers and their clients [5]; Mangshi, Ruili, and Longchuan counties in Dehong Prefecture were also included in the study. Patients were enrolled into the China CARES program in Dehong Prefecture if they met the following criteria: the World Health Organization (WHO)'s clinical stage III or IV, CD4 cell count <200 cells/μL, and ≥18 years of age. Patients were treated following the national guidelines [6] using one of four ARV regimens: AZT + 3TC + nevirapine (NVP), stavudine (D4T) + 3TC+ EFV, D4T + 3TC + NVP or didanosine (DDI) + 3TC + NVP. After initiating ARV therapy, patients were scheduled to have subsequent clinic visits at intervals of 2 weeks and 1, 2, 3, 6, 12, 18 and 24 months.

The HIV epidemic in Wenxi County of Shanxi Province was caused by nonhygienic plasma collection [7]. Wenxi County is the site of China CIPRA Subproject 4. China CIPRA Subproject 4 is a single-arm, open-label clinical trial to evaluate the impact of ARV treatment on the progression of HIV. The eligibility criteria for participating in the trial were as follows: WHO's clinical stage III or IV, CD4 cell count <350 cells/μL and ≥18 years of age. The ARV regimen was combivir (AZT+3TC) + EFV. Patients were scheduled for follow-up clinic visits at weeks 1, 2, 3, 4, 8, 12, 24, 36, 48 and 52 after initiating ARV treatment.

The costs incurred in the first year of HAART were difficult to determine because of the range of signs and symptoms of AIDS. Therefore, participants who had not received HAART at the time of enrollment were included in our analysis. The eligible patients were screened and enrolled at Mangshi, Ruili and Longchuan hospitals as designated by the Dehong Prefecture Health Bureau in the China CARES program, and at Wenxi County Hospital Clinic as designated by China CIPRA.

### Data Collection

Data were collected from January to June 2007 in designated hospitals in the four participating counties. Because of the limited budget for our research, patients who had integrated treatment expenditure materials in China CARES were randomly selected based on their treatment identification numbers in Dehong Prefecture. Two researchers were trained to review medical records and billing files using predesigned data collection forms. The following data were collected: demographics (age, gender, marital status, ethnic group, level of education, occupation, residential status and drug use), baseline CD4 cell count, date of antiretroviral medication (ARV) initiation and termination, ARV regimen, adverse drug events (ADEs), OIs and itemized costs. Expenditure data were collected starting from patient entry to ARV treatment until the end of the first year in Yunnan and Shanxi. Our study was approved by the Institutional Review Board of the Institute of Pathogen Biology, Chinese Academy of Medical Science. Written informed consent was provided from all patients when they initiated HAART.

ARV drugs are directly managed by the Provincial Center of Disease Control and Prevention or Medical Politics Division and distributed to the designated hospital. Thus, it is easy to calculate the cost of ARV drugs. However, there is no consistent data management system for Chinese medical insurance. Data collection in our study required substantial effort. To retrieve accurate expenditure data, we printed every patient's bill from the designated hospital financial management computer system. All items in the bills were categorized according to the diagnosis of OIs and ADEs. All patients who needed hospitalization were referred to a county-level hospital within our study sites. Therefore, we were able to obtain all inpatient expense data from the designated hospital. Outpatient expenses for ADEs or OIs at designated hospitals were also obtained in the same manner. Outpatient expenses for ADEs or OIs at township hospitals or village clinics were calculated by the designated hospital. We also separated these expenses according to diagnosis. All the items were classified as follows: medications, laboratory testing and ancillary examinations and medical service charges. The price of every item was based on the medical charge standard established by the local health bureau. The outpatient and inpatient expenses were averaged by the number of patients who experienced ADEs or OIs.

### Calculation of expenditures

The expenditures during the first treatment year were included for analyses, which were divided into four parts: (1) ARV medications; (2) other medications for prophylaxis and treatment of ADEs or OIs; (3) laboratory tests at baseline and follow-up for monitoring of ARVs safety and efficacy, such as CD4 and CD8 cell counts, hematologic parameters, chemistries, pregnancy testing, liver function tests, amylase activity, creatine kinase level, urinalysis and radiologic examinations (chest X-ray and abdominal ultrasound); and (4) other items, including nursing, infusions, bed use, counseling, blood transfusions and medical consumer goods.

According to the national treatment guidelines for China CARES and the protocol of China CIPRA Subproject 4, hematologic parameters, chemistries, pregnancy test, liver function tests, amylase activity, and creatine kinase levels are used for monitoring ARV drug safety, and CD4 cell count testing is used for monitoring treatment efficacy. HIV viral load measurement is not recommended because of the lack of laboratory capacity in the majority of geographic areas covered by China CARES. To reflect the actual cost under the national policy, we did not calculate the costs of HIV viral load tests.

All expenditures were calculated in US dollars (US$1 = 7 Chinese Yuan [CNY]). For ARV medications, expenditures were calculated on the basis of negotiated prices between provincial health bureaus and participating pharmaceutical companies. The average daily expense of ARV regimens was calculated using the total amount of the individual drug expenditure in 1 year divided by the total number of treatment days during the same time period, adjusted for standard dosages. The expenditure data over different calendar years were adjusted with a 3% annual inflation rate, using 2004 as the comparison year.

### Statistical analysis

We analyzed the expenditures in the first year after starting ARV therapy. We calculated the means and standard deviations (SD) of the total costs, as well as itemized costs for ARV medications, laboratory testing and treatment of ADEs and OIs. We further calculated the costs stratified by study site, demographic variables and baseline CD4 cell count. The CD4 cell count was categorized into three strata as follows: <100 cells/μL, 100-200 cells/μL and ≥200 cells/μL. The Kruskal-Wallis test was used to evaluate the expenditures associated with different CD4 cell count categories. Cochran-Mantel-Haenszel χ^2 ^tests were used to compare the proportions of costs among the three strata of CD4 cell counts. The potential independent factors which may affect the total expenditure included age, gender, treatment site, marital status, ethnic group, level of education, occupation, residential status, drug use, CD4 cell count and incidence of ADEs and OIs. A multivariate linear regression model was fitted to estimate the independent factors associated with total expenditures by eliminating nonsignificant variables in a stepwise manner. Only the factors significant at a level of 0.05 (two-tailed) were reported. R/S plus software version 2.8.1 (Lucent Technologies, Murray Hill, NJ, US) was used for the analyses.

## Results

### Demographic Characteristics of Study Subjects

Two hundred seventy-six patients were included in the analysis. One hundred seventy-six patients (63.8%) were from Dehong Prefecture of Yunnan Province, including 31 patients (11.2%) from Mangshi County, 32 patients (11.6%) from Ruili County and 113 patients (41.0%) from Longchuan County. One hundred patients (36.2%) were from Wenxi County of Shanxi Province. The average age of the patients at entry into ARV treatment was 39 years (range, 18-71 years). The patients had the following characteristics: 62% (170/276) were male, 71% (197/276) were married, 61% (167/276) were majority Han ethnicity, 21% (57/276) were Jingpo ethnicity, 14% (40/276) were Dai ethnicity, 4% (12/276) were other minority ethnicities, 12% (34/276) were illiterate, 45% (124/276) had an elementary school education, 84% (232/276) were farmers and 88% (242/276) were living with their families. Sixty-four patients (23.6%) had a history of drug use. The means of CD4 cell count to the time ARV treatment initiated was 181 ± 93 cells/μL (Table 1).

**Table 1:** Demographic characteristics of rural Chinese AIDS patients at entry to antiretroviral therapy, 2004-2006 (*N *= 276)

Characteristics	Number of Patients (%)
Treatment site	
Mangshi County, Yunnan Province	31 (11.2)
Ruili County, Yunnan Province	32 (11.6)
Longchuan County, Yunnan Province	113 (41.0)
Wenxi County, Shanxi Province	100 (36.2)
Age (yr)	
<30	44 (15.9)
30-44	162 (58.7)
≥45	70 (25.4)
Gender	
Male	170 (61.6)
Female	106 (38.4)
Marriage status	
Single	27 (9.8)
Married	197 (71.4)
Others	52 (18.8)
Ethnics	
Han	167 (60.5)
Jingpo	57 (20.6)
Dai	40 (14.5)
Others	12 (4.4)
Level of education	
Illiteracy	34 (12.3)
Elementary School	124 (44.9)
Junior Middle School	84 (30.4)
≥Senior Middle School	34 (12.3)
Occupation	
Farmers	232 (84.1)
Others	44 (15.9)
Live with family members	
No	34 (12.3)
Yes	242 (87.7)
Drug abuse	
No	211 (72.5)
Yes	65 (23.5)
Antiretroviral regimens	
AZT+3TC+NVP	57 (20.6)
D4T+3TC+EFV	19 (6.9)
D4T+3TC+NVP	65 (23.5)
DDI+3TC+NVP	35 (12.7)
Combivir+EFV	100 (36.2)
Baseline CD4 cell counts (cells/μL)	
<100	57 (20.6)
100-199	103 (37.3)
≥200	116 (42.0)

### ARV Treatment and Compliance

Eighty-five percent of patients (235/276) complied with ARV treatment for 1 year, and 85% of patients (235/276) remained in treatment or obtained prescriptions. Five ARV regimens were used by the patients, as follows: combivir + EFV (36% [100/276]), D4T + 3TC + NVP (23% [65/276]), AZT + 3TC + NVP (21% [57/276]), DDI + NVP + 3TC (13% [35/276]) and D4T + 3TC + EFV (7% [19/276]).

#### Total expenditures

The mean expenditure per patient during the first year after initiating ARV therapy was US$2,633 (range, US$226-US$8,679). The total expenditure for all 276 patients was US$726,847; 85.1% of the total expenditure was spent on ARV medications, 2.5% on medications for treating ADEs, 1.8% on medications for treating OIs, 7.6% on ARVs laboratory monitoring, 0.4% on laboratory tests for diagnosing ADEs, 0.4% for diagnosing OIs, 1.1% on other medical services related to ADEs and 1.0% on other medical services related to OIs (Table 2).

**Table 2:** Expenditures for care of rural Chinese AIDS patients in their first year of antiretroviral therapy (US dollars) (*N *= 276)

Expenditure item	Calculation formula	Total expenditures (%)	Average per capita expenditures
ARV medications	(Daily consultative price of ARV regimen) × (days taken actually)	$618,832 (85.1)	$2,242
Inpatient and outpatient medications for treating ADEs	(Daily wholesale price of medication for OIs or ADEs) × (prescribed dosage)	$18,151 (2.5)	$66
Inpatient and outpatient medications for treating OIs		$12,383 (1.8)	$45
ART laboratory monitoring		$55,281 (7.6)	$200
Inpatient and outpatient laboratory tests for diagnosing ADEs	Wholesale price for reagent, equipment depreciation, service charge, charges for use of space, water, electricity	$2,588 (0.4)	$9
Inpatient and outpatient laboratory tests for diagnosing OIs		$2,909 (0.4)	$11
Inpatient and outpatient medical service charges for treating ADEs		$8,964 (1.1)	$32
Inpatient and outpatient medical service charges for treating OIs	Local reference price for items of medical service	$7,739 (1.0)	$28
Total		$726,847 (100.0)	$2,633

ARV, antiretroviral; ART, antiretroviral therapy; ADEs, adverse drug events; OIs, opportunistic infections.

**Figure 1:**
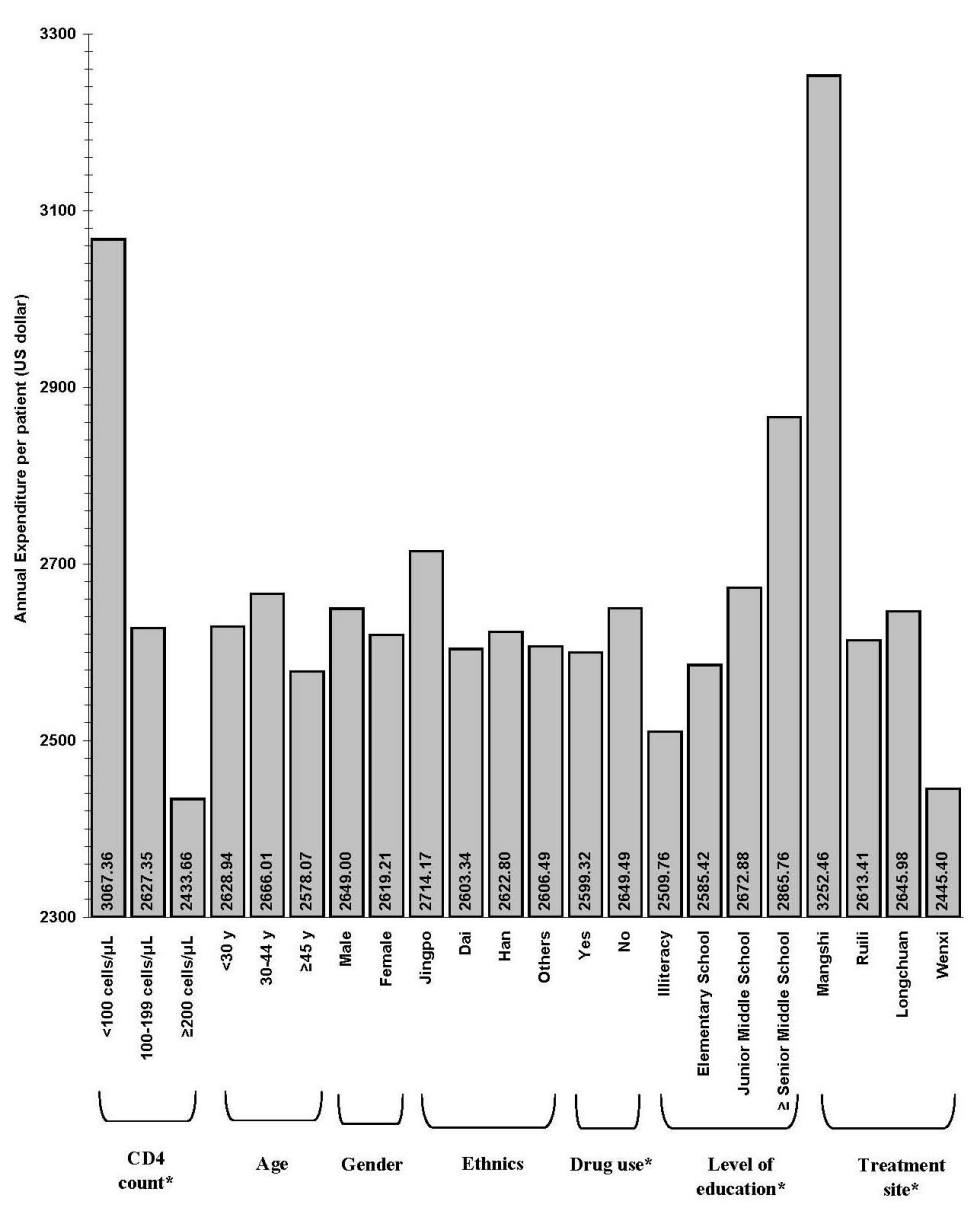
**Total expenditure among rural Chinese patients in their first year of antiretroviral therapy**. *P < 0.05. *N *= 276.

The per capita expenditures were inversely associated with CD4 cell count (*P *< 0.01). On average, patients in Mangshi had higher expenditures than patients at the other three sites (*P *< 0.01). Non-drug users had greater expenditures than drug users (*P *< 0.01). The total expenditure was also associated with the level of education, but not associated with age, gender and ethnicity (Figure 1).

### Expenditures on ARVs and safety and efficacy monitoring

A total of US$618,832 was spent on ARVs among the 276 patients in the first year of ARV therapy. The total expenditure on regular laboratory tests was US$55,281 for all patients, and the per capita expenses were US$200 (range, US$143-US$313). The total expenditure on CD4 cell count monitoring, as a part of regular laboratory tests, was US$39,429 for all patients (Table 2).

### Adverse drug event-associated expenditures

In the first year of ARV therapy, 65.6% of the patients (181/276) had at least one documented ADE. The most common ADEs were hepatotoxicity (41.3%), allergic dermatitis (23.9%), gastrointestinal disorders (21.7%) and anemia (10.5%; Table 3).

**Table 3:** Adverse drug events (ADEs) and opportunistic infections (OIs) associated expenditures among 276 Chinese patients in their first year of ARV therapy

Categories	No. of events (%)	Average expenditures (US dollars)			
		
		Medications	Tests for diagnosis	Other medical services	Total
*ADEs*					
Hepatotoxicity	114 (41.3)	$82	$12	$34	$127
Allergic dermatitis	66 (23.9)	$41	$5	$26	$72
Gastrointestinal disorders	60 (21.7)	$36	$6	$20	$62
Anemia	29 (10.5)	$43	$17	$34	$95
Dizziness and tiredness	14 (5.1)	$26	$1	$9	$36
Peripheral neuritis	5 (1.8)	$429	$11	$206	$645
Others	12 (4.4)	$16	$0	$3	$19
*OIs*					
Fungal Infection	17 (6.2)	$88	$11	$59	$158
Respiratory infection	65 (23.6)	$52	$9	$24	$85
Tuberculosis	7 (2.5)	$128	$48	$70	$245
Diarrhea	20 (7.3)	$34	$4	$20	$58
Herpes simplex virus and zoster infection	13 (4.7)	$76	$5	$26	$107
Cytomegalovirus infection	5 (1.8)	$763	$96	$349	$1,208
Others	11 (4.0)	$101	$107	$200	$408

The total expenditure for treating all ADEs was US$29,703. The medical service expenditure per person for ADEs in the first year was US$645 for peripheral neuritis, US$127 for hepatotoxicity, US$95 for anemia, US$72 for allergic dermatitis, US$62 for gastrointestinal disorders and US$36 for dizziness and fatigue (Table 3).

The medications used to treat peripheral neuritis were particularly expensive (US$429 per patient per year). Huganpian and Gansukeli (two types of traditional Chinese medicine), diammonium glycyrrhizinate (capsules or injection) and glucurolactone tablets are commonly used in China to treat hepatotoxicity, with average expenses of US$4.21 per patient per month. The average expenditure of diagnosing ADEs ranged from US$0-US$17, and other inpatient and outpatient medical service charges ranged US$3-206, including medical care for peripheral neuritis (Table 3).

No significant difference was observed in the incidence of ADEs as a function of the CD4 cell count (*P *= 0.32). Patients with lower CD4 cell counts had significantly higher expenditures for medications (*P *= 0.04), tests for diagnosing ADEs (*P *= 0.04), inpatient and outpatient medical services (*P *= 0.01) and total medical services (*P *= 0.04) compared to patients with higher CD4 cell counts (Table 4).

**Table 4:** Adverse drug events (ADEs) and opportunistic infections (OIs) associated expenditures among rural Chinese patients, stratified by CD4 cell count (*N *= 276)

Disease	CD4 cell count (cells/μL)	Prevalence (%)	Expenditures (US dollars)			
			
			Medications	Tests for diagnosis	Other medical services	Total
ADEs	<100	64.9 (37/57)	$92	$14	$37	$142
	100-199	71.9 (73/103)	$79	$11	$48	$138
	≥200	61.2 (71/116)	$42	$6	$16	$64
	*P value*	*0.32*^a^	*0.04*^b^	*0.04*^b^	*0.01*^b^	*0.04*^b^
OIs	<100	66.7 (38/57)	$138	$37	$99	$274
	100-199	57.3 (59/103)	$30	$6	$14	$51
	≥200	19.0 (22/116)	$12	$1	$5	$19
	*P value*	*<0.001*^a^	*<0.01*^b^	*<0.01*^b^	*<0.01*^b^	*<0.01*^b^

^a^Cochran-Mantel-Haenszel χ^2 ^test.

^b^Kruskal-Wallis test.

### Expenditures on OIs

Expenditures for AIDS-related OIs that occurred after initiating ARV treatment were also reviewed. Among the 276 patients, 43.84% (121) had at least one diagnosed OI in the first treatment year. The most frequent OIs were respiratory infections (23.6%), diarrhea (7.3%), fungal infections (6.2%), herpes simplex virus and zoster infections (4.7%), tuberculosis (TB; 2.5%) and cytomegalovirus (CMV) infections (1.8%; Table 3).

The total expenditure for treatment of all OIs in the first treatment year was US$23,031. The median of all medical service expenditures per person for OIs in the first year were as follows: CMV, US$1,208 (acyclovir, ganciclovir and foscarnet); tuberculosis, US$245 (isoniaizid, rifampin, pyrazinamide, ethambutol and streptomycin); fungal infections, US$158 (fluconazole, itraconazole and amphotericin B); herpes simplex virus and zoster infections, US$107 (acyclovir, ganciclovir and foscarnet); bacterial respiratory infections, US$85 (oxacillin, efoperazone/sulbactam and clindamycin); and diarrhea, US$58 (Table 3). The high cost of treating CMV was largely due to high expenses associated with medications (median, US$763) and hospitalization expenditures (median, US$349).

Those patients with lower CD4 cell counts were more likely to have at least one OI compared to those with higher CD4 cell counts (*P *< 0.01). The OI-related expenditures among patients with lower CD4 cell counts were significantly higher for medications (*P *< 0.01), diagnosing OIs (*P *< 0.01), inpatient and outpatient medical services (*P *< 0.01) and total medical expenditures (*P *< 0.01) compared to patients with higher CD4 cell counts (Table 4).

### Total expenditures and associated factors

Finally, multivariate linear regression analysis indicated that the following variables were independently associated with the total expenditure in the first ARV treatment year: CD4 cell count (100-199 cells/μL versus <100 cells/μL: *P *= 0.02; and ≥200 cells/μL versus <100 cells/μL: *P *< 0.004), residence in Mangshi (*P *< 0.0001), ADE incidence (*P *= 0.04) and OI incidence (*P *= 0.02; Table 5).

**Table 5:** Factors associated with total expenditure among rural Chinese patients in their first year of ARV therapy (*N *= 276)

Factors	Coefficient	Standard error	*F *value	*P *value
CD4 cell counts (cells/μL)				
100-199 vs. <100	-280.3	121.0	5.37	0.02
(≥200 vs. <100)	-336.4	128.0	8.20	0.004
Treatment site				
(Mangshi vs. others)	582.5	141.3	17.0	<0.0001
Adverse drug event				
(Yes vs. no)	186.4	89.5	4.3	0.04
Opportunistic infection				
(Yes vs. no)	233.1	95.0	6.0	0.02

## Discussion

This is the first study to determine the expenditure data for AIDS treatment in rural areas of China. During the first year, the average annual expenditure for AIDS treatment per person was US$2,633. Expenditures were significantly higher for patients with low CD4 cell counts compared to those with high CD4 cell counts (*P *< 0.01). The average expenditures for the treatment of ADEs and OIs increased significantly among patients with low CD4 cell counts. Therefore, initiating HAART to patients with higher CD4 cell counts appears to be effective in controlling AIDS treatment-related expenses for ADEs and OIs.

Although funding for HAART has expanded in recent years, the resources are still limited and costs typically increase with time. On the basis of our research, the expenses for ARV medications were the largest component of AIDS treatment. Among first-line agents, only AZT, D4T, DDI and NVP are generic ARVs produced by pharmaceutical companies in China, while EFV is purchased by the Chinese central government. Although 3TC was donated by GlaxoSmithKline, we still added its price into the total expenses to show the actual expenditures for ARVs. The high cost of ARVs has been the main obstacle for China in considering free second-line agents. Indinavir without a booster, Kaletra (lopinavir/ritonavir), tenofovir, emreicitabine and abacavir are used in cases of drug resistance. Some of these ARVs are now available as cost-free treatment. Merito *et al. *[8] showed that the average annual cost of ARVs increased with the duration of treatment, while inpatient costs decreased. Bruno *et al. *[9] showed the total expense shifted from inpatient costs to ARV medication expenses. China will also face this same issue, along with the progression of HIV infection and the increasing number of AIDS patients in the near future. Duraisamy *et al. *[10] showed that the median direct cost of treatment and services was US$610 for 6 months in south India for patients who received HAART in 2001. According to the report from the CDC center of the Health Ministry of Iran [11], the direct cost of HIV treatment was US$1,025 per person in 2005. A Philippines study [12] reported that during the early symptomatic stage, the cost per case per year was US$301, and in the late symptomatic stage the cost increased to US$5,774 in 1996. Compared with these studies, the mean expenditure in this study was a little higher because many patients in the last symptomatic stage received HAART at the inception of China CARES. A greater number of AIDS patients receive HAART because of free ARVs and reimbursement for laboratory tests and transportation. Thus, some insurance policy should be developed to lower the patients' financial burden and promote compliance.

A higher level of education was associated with a higher cost of HAART-related expenses in our study, which was consistent with the results of Bozzette *et al. *[13]. In rural China, patients with a higher level of education may receive more information about AIDS and related health care policies. Therefore, they may use more health services than patients with a lower level of education.

ADEs contribute significantly to HIV/AIDS treatment costs, which can be mitigated through early symptom assessment and management. The ADEs associated with ARVs have been reported in many studies [14-20]. Besides financial constraints, ADEs are also important factors which affect HAART compliance [21-23], but few studies have determined the monitoring and treatment expenses of ADEs [24]. We explored the expenditures for ADEs in our study and showed that the expenditures for the treatment of peripheral neuritis were the highest because of high costs of medications. Hepatotoxicity was the most common AIDS-related ADE, thus regular laboratory monitoring for hepatotoxicity in many ARV regimens is standard. NVP was more likely to induce hepatotoxicity, which is consistent with studies in China and other countries [14-20]. Expenditures for the treatment of anemia were the third highest among the ADEs. The main components of the increased expenditures for the treatment of anemia were blood transfusion and hospitalization expenditures. More attention should be paid to regular monitoring of the blood cell count. Early detection and early treatment will decrease the expenditure of hospitalization.

HAART has been shown to be effective in increasing the CD4 cell count and reducing the incidence of OIs [25-27]. In the current study, OIs still occurred in some patients who received ARVs. The expenditures for some OIs with a lower incidence, such as CMV and fungal infections, were higher than the treatment costs of other OIs. Long-term hospital stays and higher expenditures for antiviral and antifungal drugs and special laboratory tests result in higher expenditures for OIs. HIV/TB coinfections deserve particular attention because of the high prevalence and cost. The treatment of HIV/TB coinfection was the second highest expenditure of all OIs in this study. China has the second-highest prevalence rate of TB in the world. According to a WHO report [28], the incidence of TB increased at an average rate of 7% per year in those countries with a high prevalence of HIV (≥5%), but the average rate of increase was only 1.3% in countries with a low prevalence (≤5%). HIV-infected patients are at a markedly increased risk for progressive disease following primary TB infection [29-31]. Thus, screening and treatment of TB at the time of HIV infection is beneficial to patients and improves the efficacy of HAART. With respect to the optimal time to initiate HAART, it is worth noting that the immune reconstitution syndrome tends to be worse in patients initiating HAART with lower CD4 cell counts. Some of the patients in our study were in the last symptomatic stage, which may increase the incidence of OIs and sequentially increase the expenditure for treatment.

In this study, we also showed that the incidence of *Pneumocystis carinii *pneumonia (PCP) was very low and may be attributed to regular prophylaxis using sulfamethoxazole. Because the sample size was limited in our study, the correlation between the use of sulfamethoxazole for prophylaxis and the incidence of PCP should be the subject of study in China.

When to begin HAART is an important issue which has been much discussed in recent years. Studies in high-income countries have verified that initiating HAART in patients with higher CD4 cell counts can improve the efficacy of treatment [32]. However, for resource-limited countries, where viral load testing is often not available for the diagnosis of AIDS, the CD4 cell count and symptoms are the main indicators for initiating HAART. As a result, some patients are not diagnosed in a timely manner. A lower CD4 cell count was associated with higher treatment expenditures. This effect has also been shown in patients with CD4 cell counts <50/μl in other studies [13,33]. In our study, we noted a similar result, although the sample size was limited. Over the long term, initiating HAART to patients with higher CD4 cell counts is likely to effectively control AIDS treatment expenses for ADEs and OIs, but it will also increase the treatment costs over a longer period of treatment. Thus, factors, such as the financial burden of patients, care policy and compliance should be generally considered when initiating HAART.

In our study, we pooled the data from two distinct programs, the only difference in program design of which was the enrollment criteria for CD4 cell counts. However, the CD4 cell count was similar in the two programs after considering the WHO's clinical stages. Pooling data can increase the sample size and yield more information about HAART expenditures. Moreover, the price of medications and medical services was similar in the different sites of the program, although the different frequencies of follow-up may produce heterogeneity in expenses (that is, a greater number of follow-up visits in the China CIPRA).

The main limitation of the study was that the results cannot be extended to all of China because of the differences in the cost of living across China [34]. Second, Chinese public hospitals are classified into three levels according to the national hospital administration standard. Higher-level hospitals had more choice of medications, ancillary examinations and higher medical service prices. In our study, Mangshi Hospital was the highest-level hospital and was associated with a higher cost of HAART-related expenses. However, other AIDS treatment expense data were from county-level hospitals, which were usually second-level hospitals. Thus, our results are likely to have underestimated the costs of hospital care compared with the highest-level hospitals in cities and overestimate the costs compared with township hospitals and village clinics. Third, our research did not address the opportunity cost, expenses outside the hospital system and the association between compliance and cost because of the difficulty of collecting data.

## Conclusions

Despite the above limitations, this study is the first to provide important information on the cost of rural AIDS patients in China. The ARVs expense was the largest part of HAART cost, averaging US$2,242 per patient in the first ARV treatment year. These costs appear to increase over time in China, as consequences of the increasing number of possible occurrences of drug resistance in the continuing period of HAART and more second-line regimens are used. Over the long term, initiating HAART in patients with a higher CD4 cell count is likely to effectively control AIDS treatment expenses on ADEs and OIs, but some factors should be generally balanced when initiating HAART.

## List of abbreviations

ADEs: adverse drug events; AIDS: acquired immune deficiency syndrome; ARV: antiretroviral; ARVs: antiretroviral medications; AZT: zidovudine; China CARES: China Comprehensive AIDS Response; CMV: cytomegalovirus; CNY: Chinese yuan; D4T: stavudine; DDI: didanosine; EFV: efavirenz; HAART: highly active antiretroviral therapy; IDUs: injection drug use; IDV: indinavir; Kaletra: lopinavir/ritonavir; NRTI: nucleoside reverse transcriptase inhibitors; NNRTI: non-nucleoside reverse transcriptase inhibitors; NVP: nevirapine; OIs: opportunistic infections; PCP: *Pneumocystis carinii *pneumonia; TB: tuberculosis; WHO: World Health Organization; 3TC: lamivudine.

## Competing interests

The authors declare that they have no competing interests.

## Authors' contributions

FZ designed the project, collected data and drafted the first version of the manuscript. GFK revised the manuscript. HZQ oversaw the data analysis and revised the manuscript. SD collected laboratory data. JSW participated in data analysis. ZWG collected and analyzed data. XPZ reviewed medical records and collected data at the Wenxi site. All authors read and approved the final manuscript.

## Pre-publication history

The pre-publication history for this paper can be accessed here:

http://www.biomedcentral.com/1741-7015/9/6/prepub
